# Developing molecular surveillance of SARS-CoV-2 in the Czech Republic (2021–2022)

**DOI:** 10.1038/s41598-025-01074-3

**Published:** 2025-06-04

**Authors:** Timotej Šúri, Lucie Pfeiferová, Matěj Bezdíček, Jan Svatoň, Vladimír Hampl, Karel Berka, Helena Jiřincová, Martina Lengerová, Martin Kolísko, Alexander Nagy, Ruth Tachezy, Czech COVID-19 Genomics Consortium, Timotej Šúri, Lucie Pfeiferová, Matěj Bezdíček, Jan Svatoň, Vladimír Hampl, Karel Berka, Helena Jiřincová, Martina Lengerová, Martin Kolísko, Alexander Nagy, Ruth Tachezy, Jan Bartoš, Petr Brož, Vojtěch Bystrý, Martin Čech, Monika Čechová, Jiří Černý, Kateřina Chudějová, Iva Dolinová, Pavel Dřevínek, Edvard Ehler, Alena Fialová, Filip Franko, Viktor Furman, Zoltán Füssy, Markéta Gančarčíková, Alejandro Jiménez González, Marián Hajdúch, Blanka Hamplová, Václav Hejret, Petr Holub, Patrik Horna, Miluše Hradilová, Štěpánka Hrdá, Magdalena Jančářová, Michaela Jaroměřská, Eduard Ježo, Daniel Klimeš, Šárka Kocourková, Jana Kozáková, Martin Kracík, Jan Kubovčiak, Jana Fialová Kučerová, Jan Kynčl, Matej Lexa, Monika Liptáková, Jan Macháň, Barbora Macková, Marek Malý, Kateřina Matějová, Luděk Matyska, Hana Medová, Ondřej Moravčík, Jakub Mrázek, Serafim Nenarokov, Eva Niklova, Marian Novotný, Jaroslav Nunvář, Adam Obr, Hana Orlíková, Josef Pasulka, Helena Paszeková, Ingrid Poláková, Martin Pospíšek, Miroslav Ruda, Jana Šáchová, Eric Salomaki, Jeffrey Silberman, Radek Šíma, Branislav Šiška, Rastislav Slavkovský, Klára Sobotíková, Kateřina Štillerová, Viktor Stránecký, Alexander Tice, Boris Tichý, Markéta Tomková, Karolína Trachtová, Sebastian Cristian Treitli, Jaromíra Večeřová, David Vostřák, Jan Vrbský, Monika Wisniewska, Artsemi Yushkevich, Tomáš Zajíc, Martin Zmuda, Michal Kolář, Jan Pačes, Michal Kolář, Jan Pačes

**Affiliations:** 1 National Institute of Public Health, Prague, Czech Republic; 2 Institute of Molecular Genetics of the Czech Academy of Sciences, Prague, Czech Republic; 3 University of Chemistry and Technology, Prague, Czech Republic; 4 University Hospital Brno and Faculty of Medicine, Masaryk University, Brno, Czech Republic; 5 CEITEC Masaryk University, Brno, Czech Republic; 6 Faculty of Science, Charles University - BIOCEV, Prague, Czech Republic; 7 Department of Physical Chemistry, Faculty of Science, Palacký University in Olomouc, Olomouc, Czech Republic; 8 Biological Center of the Academy of Sciences of the Czech Republic, České Budějovice, Czech Republic; 9 State Veterinary Institute, Prague, Czech Republic; 10 Institute of Experimental Botany of the AS CR, Olomouc, Czech Republic; 11 Bioxsys s.r.o., Prague, Czech Republic; 12 Institute of Organic Chemistry and Biochemistry of the Czech Academy of Sciences, Prague, Czech Republic; 13 Faculty of Informatics Masaryk University, Brno, Czech Republic; 14 Czech University of Life Sciences, Prague, Czech Republic; 15 Faculty of Medicine in Pilsen, Charles University, Plzeň, Czech Republic; 16 Regional Hospital Liberec, a. s., Liberec, Czech Republic; 17 Second Faculty of Medicine – Charles University, Prague, Czech Republic; 18 GHC Genetics, s.r.o., Prague, Czech Republic; 19 University of South Bohemia in České Budějovice, České Budějovice, Czech Republic; 20 University Hospital Hradec Králové, Hradec Králové, Czech Republic; 21 Institute of Molecular and Translational Medicine, Olomouc, Czech Republic; 22 Institute of Computer Science Masaryk University, Brno, Czech Republic; 23 Public Health Institute Ostrava, Ostrava, Czech Republic; 24 Institute of Health Information and Statistics of the Czech Republic, Prague, Czech Republic; 25 Faculty of Medicine Masaryk University, Brno, Czech Republic; 26 University Hospital Ostrava, Ostrava, Czech Republic; 27 Institute of Hematology and Blood Transfusion, Prague, Czech Republic; 28 Ludwig Cancer Research Oxford, University of Oxford, Oxford, UK; 29 St. Anne’s University Hospital Brno, Brno, Czech Republic; 30 Institute of Microbiology of the Czech Academy of Sciences, Prague, Czech Republic

**Keywords:** SARS-CoV-2 variants, Molecular surveillance, Variant discrimination PCR, Czech Republic, Virology, Microbial genetics

## Abstract

Molecular surveillance was widely used during the COVID-19 pandemic to detect rapidly emerging variants and monitor the transmission of SARS-CoV-2 within communities. In 2021, the Czech COVID-19 Genomics Consortium (COG-CZ) was set up to coordinate a new SARS-CoV-2 molecular surveillance network. In the Czech Republic, molecular surveillance employed whole genome sequencing (WGS) and variant discrimination polymerase chain reaction (VD-PCR) on samples collected through passive, active and sentinel surveillance. All WGS data was uploaded to GISAID and the PANGO lineages used by GISAID were compared to the main variants determined by VD-PCR. To assess the effectiveness and reliability of the gathered data in adapting pandemic responses, the capabilities and turnaround times of the molecular surveillance methods are evaluated. VD-PCR results were available within 48 h of sample collection for 81.5% of cases during the Delta/Omicron transition. WGS enabled the detection of low-frequency novel variants in infection clusters. WGS surveillance showed there was community spread of AY.20.1, a variant that gained novel mutations within the Czech Republic. Molecular surveillance informed the implementation of public health measures; temporal comparisons of restrictions and outcomes are described. Further areas for improvement have been identified for monitoring and managing future pandemics.

**Supplementary Information:**

The online version contains supplementary material available at 10.1038/s41598-025-01074-3.

## Introduction

The COVID-19 pandemic had a significant impact on public health systems worldwide. The Czech Republic experienced multiple waves of infections, with over 3.8 million confirmed cases and over 30 thousand deaths between 2021 and 2022^1^. Initially, the country did not have an effective surveillance system set up, and mitigation of the pandemic was done by implementing common epidemiological measures such as travel restrictions, social distancing, and widespread testing.

A complex surveillance system needed to be developed to monitor the molecular evolution of the SARS-CoV-2 pandemic, and to ensure the detection of immunologically divergent lineages—genetically related virus groups that share a common ancestor—and variants—viral forms with distinct genetic mutations that may impact transmissibility, immune escape, or disease severity. The system consisted of a combination of passive surveillance, which relies on healthcare providers to report laboratory-confirmed cases, and active surveillance which includes targeted testing of specific populations, such as contacts of confirmed cases or individuals in high-risk settings. The system also includes sentinel surveillance, which involves the regular collection and testing of samples from selected populations to monitor the prevalence of virus lineages.

Molecular surveillance, including whole genome sequencing (WGS) and variant discrimination polymerase chain reaction (VD-PCR), enables the identification and monitoring of new viral mutations. This allows public health officials to gain insight into the characteristics of the virus, such as its transmissibility, virulence or resistance to vaccines and treatments. These data supported real-time tracking of variants, were used to inform vaccination recommendations, and accompanied changes to travel restrictions. The data analyzed in this study were generated by COG-CZ and were made available on GISAID for global monitoring and modelling efforts. Neighbouring countries could be compared based on the impact restrictions had on virus transmission.

In February 2021, COG-CZ was established to improve the accuracy, speed, and volume of genomic sequencing of the virus and analysis of its lineages. During 2021 and 2022, the national sequencing effort consisted of public health and research institutions and was financially supported by the European Centre for Disease Control (ECDC) Health Emergency Preparedness and Response Incubator (HERA) grant and the Czech Ministry of Health.

This study analyzes variant dominance transitions and describes concurrent changes in pandemic responses and excess mortality trends. We aim to review the national genomic surveillance strategy, evaluating sequencing coverage, response time and regional variant distribution, to inform future pandemic preparedness.

## Methods

### Data collection for surveillance

The data analysed originated from the Czech Information System of Infectious Diseases (ISIN) database, which is required by current legislation to record national testing, vaccination, epidemiological data, and sequencing results, which allowed for daily reporting of necessary data to all stakeholders^2^. The obtained genomic sequences and the pseudonymised epidemiological data were submitted to the GISAID and ISIN databases by COG-CZ members^2,3^. The analysis is based on metadata associated with 56,033 sequences available on GISAID up to the 20th of October 2023, via gisaid.org/EPI_SET_231101rf (Supplement 5). WGS data was exported from GISAID, and pangolin lineages determined by GISAID at the time of export were used for further analysis^3,4^. Excess mortality in the 2021–2022 period was measured as the rate of additional deaths in a week compared to the expected number of deaths in the same week over a baseline reference period (2016–2019). Mortality data in the Czech Republic is gathered by the Czech Statistical Office and is reported as weekly data.

WGS data from GISAID was used to determine variant spread in regions using Python version 3.11.7 and the pandas, matplotlib and seaborn libraries. To determine spike protein frequency using the R (version 4.3.1) packages ggplot2, cowplot, ggpubr, and sf.

### Sample selection

Sample selection for sequencing was determined by an ISIN algorithm that recognised six key risk indicators. Samples from cases imported from a high-risk country or region, cases from clusters of infection, reinfection, vaccination breakthrough infections, unusual sample types (tissue, blood, plasma), and hospitalisation with severe disease in young or otherwise healthy patients were highlighted to testing laboratories in daily emails from ISIN with instructions to ship the samples to their assigned sequencing centre. Travel history was filled in by patients using an online form on arrival while the remaining categories were determined automatically by the ISIN algorithm once positive PCR test results were uploaded, along with the risk profile of the patient created by the examining physician. The central ISIN database contained the vaccination and infection records for all patients. The categories were weighted equally in the selected samples, therefore as the proportion of reinfection and vaccine breakthrough infections rose, a higher overall proportion of samples from rarer categories such as import, clusters and severe disease would be sequenced when they occurred. Sentinel laboratories also shipped a collection of arbitrarily selected positive samples.

Sequencing was performed in ten core sequencing centres assigned to sentinel laboratories in each region based on proximity, ensuring samples were collected to cover the whole Czech Republic (Supplementary Fig. 1). Each sequencing centre collected representative samples for one or two of the fourteen administrative regions of the Czech Republic, for an approximate coverage of 1 million inhabitants per sequencing centre (Supplement 2). Confirmed cases were attributed to regions using the postcode of their permanent address or address filled in through a case identification form requested by Institute of Health Information and Statistics of the Czech Republic (IHIS/UZIS).

### Variant discrimination PCR assay

Between the 1st of July 2021 and the 31st of January 2022, the testing of all positive samples by VD-PCR was mandated and financed by national health insurance companies. Testing laboratories were also advised to test samples using VD-PCR as early as May 2021. They received regular updates on the recommended mutation composition the testing kits should cover by the National Reference Laboratory (NRL) throughout 2021 and 2022.

VD-PCR assay kits consist of PCR probe-based detection of specific mutations in the spike gene. The NRL recommended testing for at least two mutations in the spike protein, E484K and L452R, with a recommendation to also test for N501Y, K417N and P681H. The composition of mutations detected by commercial kits varied; however, the kits that also tested the spike mutations Del69-70, T478K and S477N were recommended (Supplement 3). These mutations were selected to enable discrimination of the variants; however, the Beta and Gamma variants could not be differentiated by E484K and L452R, and would need to test for K417N, which some kits did not contain at the time. The Alpha and Omicron variants would similarly need K417N, Del69-70, T478K or S477N to differentiate them, though these variants did not co-occur.

Mutations detected by VD-PCR assays were interpreted centrally by ISIN to determine the predicted main variant. Results of VD-PCR assays were requested from IHIS/UZIS for the 2021–2022 period^2^. WGS data was used to confirm or correct the resulting variant.

### Whole genome sequencing

Sequencing centres used Illumina, Oxford Nanopore, and Ion Torrent systems. For Illumina sequencing, whole genome libraries were prepared using NEBNext ARTIC (New England BioLabs) or CleanPlex SARS-CoV-2 (Paragon Genomics) workflows. ARTIC^5^ amplicon-based libraries were used for Oxford Nanopore sequencing within the RAMPART^6^ workflow. The Genexus Ion Torrent system was used for low capacity, fast turnaround WGS. Data produced as part of the ECDC sequencing support service was also generated using the ARTIC workflow by Eurofins and processed using an in-house pipeline. Most samples were prepared using amplicon-based library-preparation kits and needed to ensure that updated primers were used to prevent amplicon dropout, particularly during the transition from Delta to Omicron.

WGS data was analysed using unique pipelines for each library preparation method. Illumina NEBNext data was processed using a modified nf-core/viralrecon v1.1.0 pipeline^7^. Raw FASTQ files were merged, quality-checked with FastQC, and trimmed with fastp (mean quality cutoff 20, Phred cutoff 20, minimum length 50). Amplicon data were processed using the varskip2 scheme—with specific FASTA and BED files—and enforced a 65 bp insert size while analyzing positions 46–29,799 against the Wuhan-Hu-1 reference genome (NC_045512.2). Reads were mapped with BWA^8^ and processed with SAMtools, with primer removal performed by iVar trim (offset 0, end offset 2, minimum length 30) and optional duplicate marking via Picard. Variant calling was carried out using Freebayes^9^, with subsequent annotation by SnpEff/SnpSift and consensus quality assessment by QUAST. Workflow reporting was integrated with MultiQC, and while steps for host read removal (Kraken2) and de novo assembly (via SPAdes, Unicycler, or minia) were available, they were skipped in this configuration. Data from libraries prepared using CleanPlex SARS-CoV-2 kits were analysed using commercial software (Bioxsys). Data generated using Oxford Nanopore systems was analysed using an in-house pipeline (unpublished) or the ARTIC bioinformatics platform. The Ion Torrent system uses an automated proprietary pipeline for the research SARS-CoV-2 panel. High-quality sequences were uploaded to GISAID (Supplement 4).

For monitoring purposes, the ECDC recommends that the sequencing sample size be set dynamically to allow the detection of a novel variant circulating in 1.0–2.5% of confirmed cases, which was calculated to require sequencing 273 to 1522 samples per week depending on the number of cases^10^. The target for Czech WGS surveillance was set to 70–100 samples selected by ISIN per week per region totalling 980–1400 samples. To provide actionable information to guide public health policy, WGS data must be current and provide information about variant circulation in samples no older than 14 days and ideally less than 7 days after sample collection. Each of the 10 sequencing centres maintained the capacity to sequence 50–200 samples per week and received semi-regular shipments of samples from testing laboratories and sentinel laboratories from across their regions.

## Results

### Overview

**Fig. 1:**
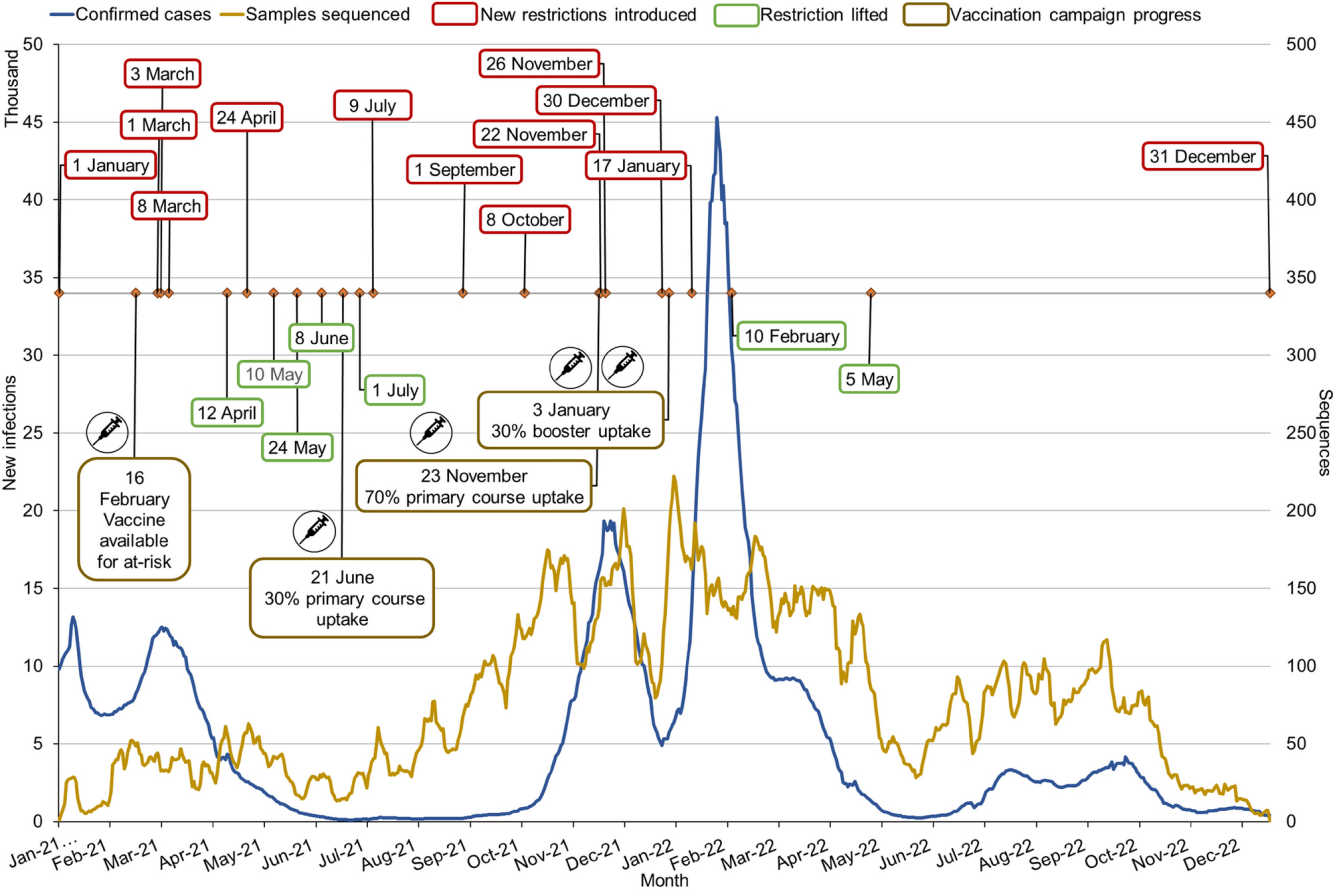
Progression of the COVID-19 pandemic in the Czech Republic in 2021 and 2022. The timeline of pandemic restrictions (Supplementary Table 3) and key points of the vaccination campaign are overlaid over the 7-day running average of daily confirmed cases (blue). Newly introduced restrictions are shown as dates with red outlines, while the lifting of specific restrictions is shown as dates in green, with notable points of the vaccination campaign in yellow. Descriptions of these restrictions are found in Supplementary Table 3. The 7-day running average number of samples sequenced (green), by date of collection, shows the available sequencing capacity as the sequencing network was formalised and came online in 2021.

The data describes three pandemic waves and two periods of government state of emergency, examining the transitioning of variants during the Alpha, Delta and Omicron waves (Fig. 1). A national genomic surveillance policy was developed and implemented during this period, and a SARS-CoV-2 surveillance network was established. Between January 1, 2021 and December 31, 2022, there were 3,843,207 cases of laboratory-confirmed COVID-19 reported in the Czech Republic, of which 1,159,145 had a VD-PCR assay performed, and 56,033 samples were sequenced and uploaded to GISAID, corresponding to 30.1% and 1.5% of confirmed cases respectively.

Surveillance of variants circulating in the Czech Republic

**Fig. 2:**
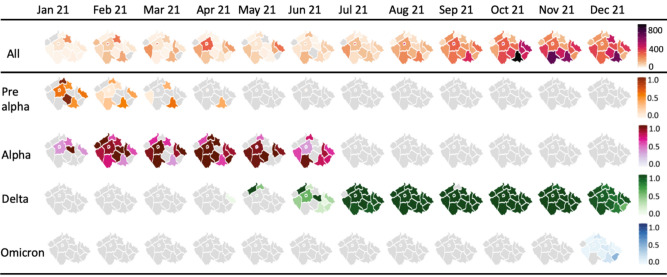
Spatiotemporal spread of SARS-CoV-2 variants of concern within the Czech Republic between January and December 2021. The main variants shown are Pre-alpha (B.1.258 and similar), Alpha (B.1.1.7), Delta (B.1.617.2 or AY.X), and Omicron (BA.X), in monthly intervals. The first row shows the total number of positive cases in the regions according to GISAID. The bottom figure shows the frequencies of each variant in the regions.

A generally balanced distribution of sequenced samples in relation to the geographic distribution of the Czech Republic’s population was achieved, as seen in Fig. 2. The spread of new variants replacing currently circulating ones within weeks highlights the interconnectedness of the regions.

**Fig. 3:**
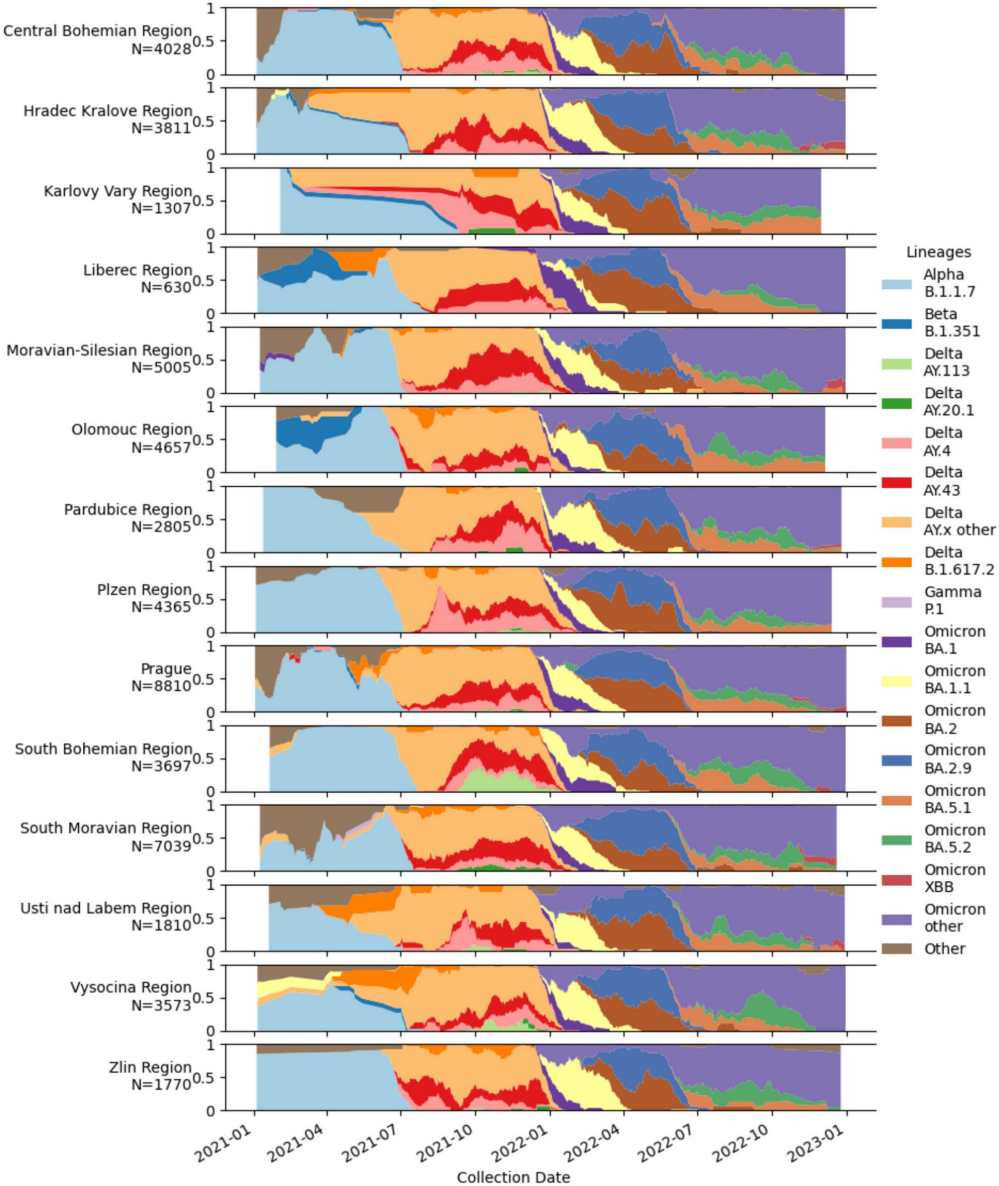
Relative distribution of SARS-CoV-2 lineages in the regions of the Czech Republic from January 2021 to December 2022. The relative prevalence of the most abundant SARS-CoV-2 lineages and changes in their distribution over time and across the regions of the Czech Republic.

During the 2021–2022 period, the relative distribution of SARS-CoV-2 lineages within individual regions of the Czech Republic has been analysed (Fig. 3). Notably, the B.1.1.7 (Alpha), B.1.617.2 (Delta), BA.1, and BA.2 (Omicron) variants were detected at the highest frequency in all regions at similar time periods. However, localised outbreaks of specific lineages can also be seen. There were 469 cases of AY.113 identified, with the majority (n = 354; 75.5%) detected in the South Bohemian region and 20.0% (n = 94) in the adjacent Vysocina region. For AY.20.1, 165 cases were detected, of which 93.9% (n = 155) were in the South Moravian region and 6.1% (n = 10) in the adjacent Zlin region. Additionally, several less frequent SARS-CoV-2 lineages were observed localised only in specific regions of the Czech Republic, each detected in fewer than 100 cases. The main variants of SARS-CoV-2 were able to spread to all regions, however, the local spread of less common lineages emphasises the need for ongoing monitoring and implementation of control measures to prevent the spread of potentially highly virulent new lineages to other regions.

**Fig. 4:**
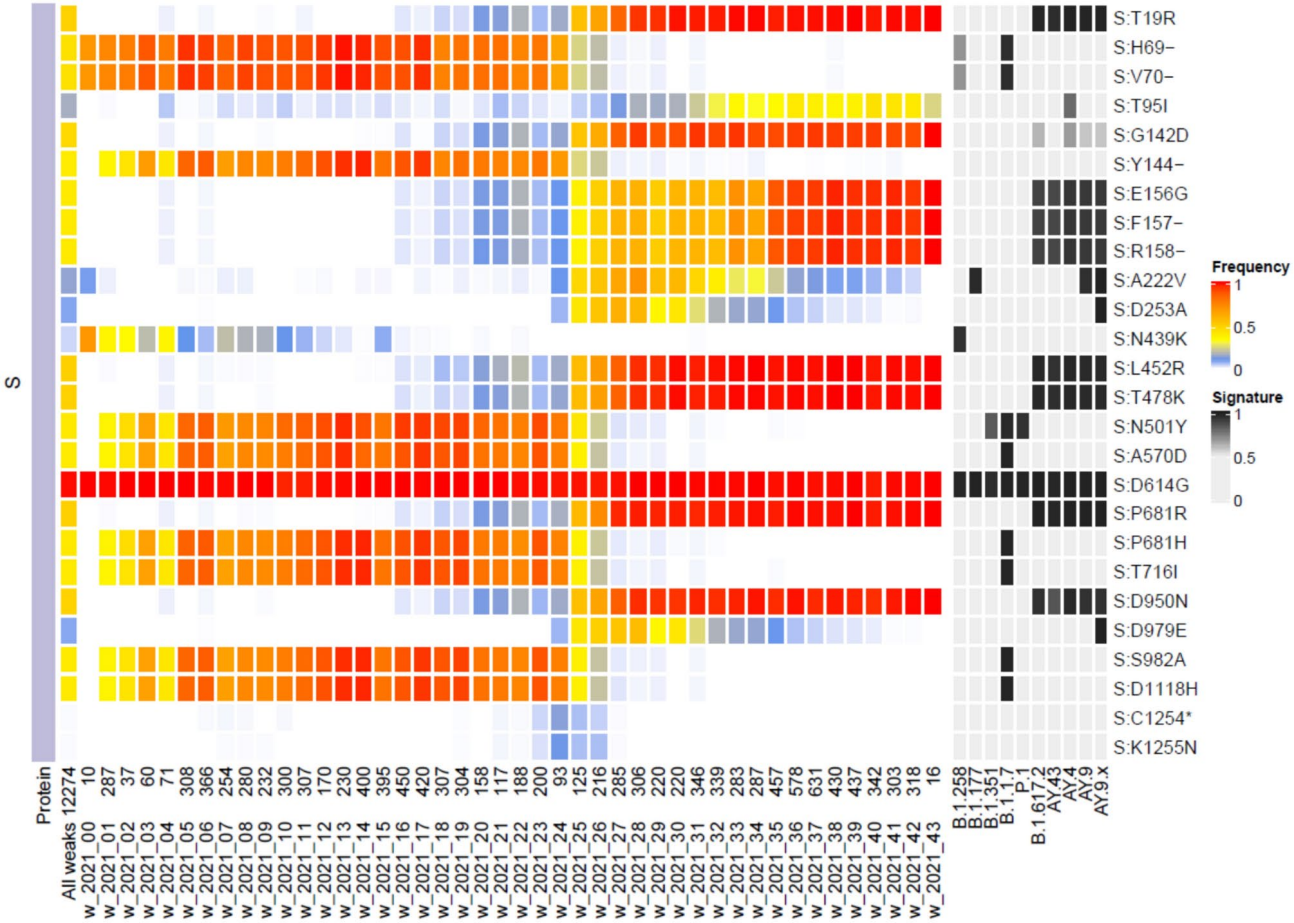
Transition from Alpha to Delta variant dominance in 2021 as seen in the frequencies of amino acid mutations in the Spike protein (S) over time. Mutations compared to reference genome NC_045512.2 occurring in more than 10% of weekly isolates where at least three samples contained the mutation. Total number of weekly samples shown on the bottom axis. Common variants and their typical mutations are shown along the right for comparison. Alpha variant frequency decreased from week 17 of 2021 as the Delta variant started spreading. The frequency of Alpha-specific mutations such as S:Del69/70, S:N501Y or S:P681H decreased while the introduction and spread of the Delta variant could be seen in the rising frequency of S:L452R, S:T478K and S:P681R.

Fortnightly reports containing detailed SARS-CoV-2 genomic variability and epidemiology analysis using GISAID data were published on https://virus.img.cas.cz/^11^. In these reports, the emergence of individual mutations and their linkage to variants was analysed. Figure 4 shows that as the Alpha variant emerged in the Czech Republic in December 2020, it co-circulated with the Central European variant B.1.258 (S:N439K) until it displaced it by week 16 of 2021. The Alpha (B.1.1.7) variant was displaced by Delta (B.1.617.2) in weeks 24 to 26, as seen in the frequency of S:Del69/70 and S:L452R. A short co-emergence of a novel mutation S:K1255N was only seen during these transition weeks. The first cases of Delta variant could therefore be spotted as early as week 16 of 2021.

### Epidemiological response

One of the aims of establishing genomic surveillance was to promptly provide data on circulating variants to public health authorities so that measures that might reduce morbidity and mortality could be implemented quickly when needed. The Alpha variant was indirectly detected in some PCR assays by the cycle threshold shift in the N gene in December 2020. From the 1st of January 2021, the government gradually implemented stricter restrictions (Supplement 6). On January 20, 2021, the first genomic sequences of the Alpha variant in the Czech Republic were published, more than two weeks after sample collection. WGS enabled the detection of the B.1.1.7 (Alpha) variant, which prompted immediate travel restrictions and enhanced monitoring of travelers from the United Kingdom. In March 2021, the Czech Republic had the highest number of COVID-19 cases per million people in the world (1190, March 5, 2021)^12^. By the end of March 2021, only 14.5% of adults had received at least one dose of a vaccine^13^.

The pandemic waves starting in November 2021 were caused by Delta and Omicron variants. The Delta variant became the dominant variant in June 2021. Emergence of the Delta variant led to refined quarantine measures and targeted interventions in high-incidence regions. Sequencing data informed vaccination strategies for high-risk populations and guided updates to vaccine compositions, as novel mutation detection predicted the increase in disease severity and immune escape of the Delta variant. From June 2021 there was an increasing focus on vaccination, with many activities requiring a vaccination pass. From October the plateau of vaccination reversed and by November, the overall vaccination rate for the primary vaccination course approached 60%^13^. On the 26th of November 2021, a new state of emergency was declared in response to rising case numbers. The resulting measures did not significantly increase pandemic restrictions, particularly for vaccinated individuals, who were largely exempt. By the end of 2021, up to 73.0% of adults had finished the primary vaccination course and 27.9% had received their first booster^13^. On the 5th of May 2022, the pandemic response effectively ended, and nearly all pandemic restrictions were lifted as the 7-day average of new cases fell below 1500.

### Excess mortality

**Fig. 5:**
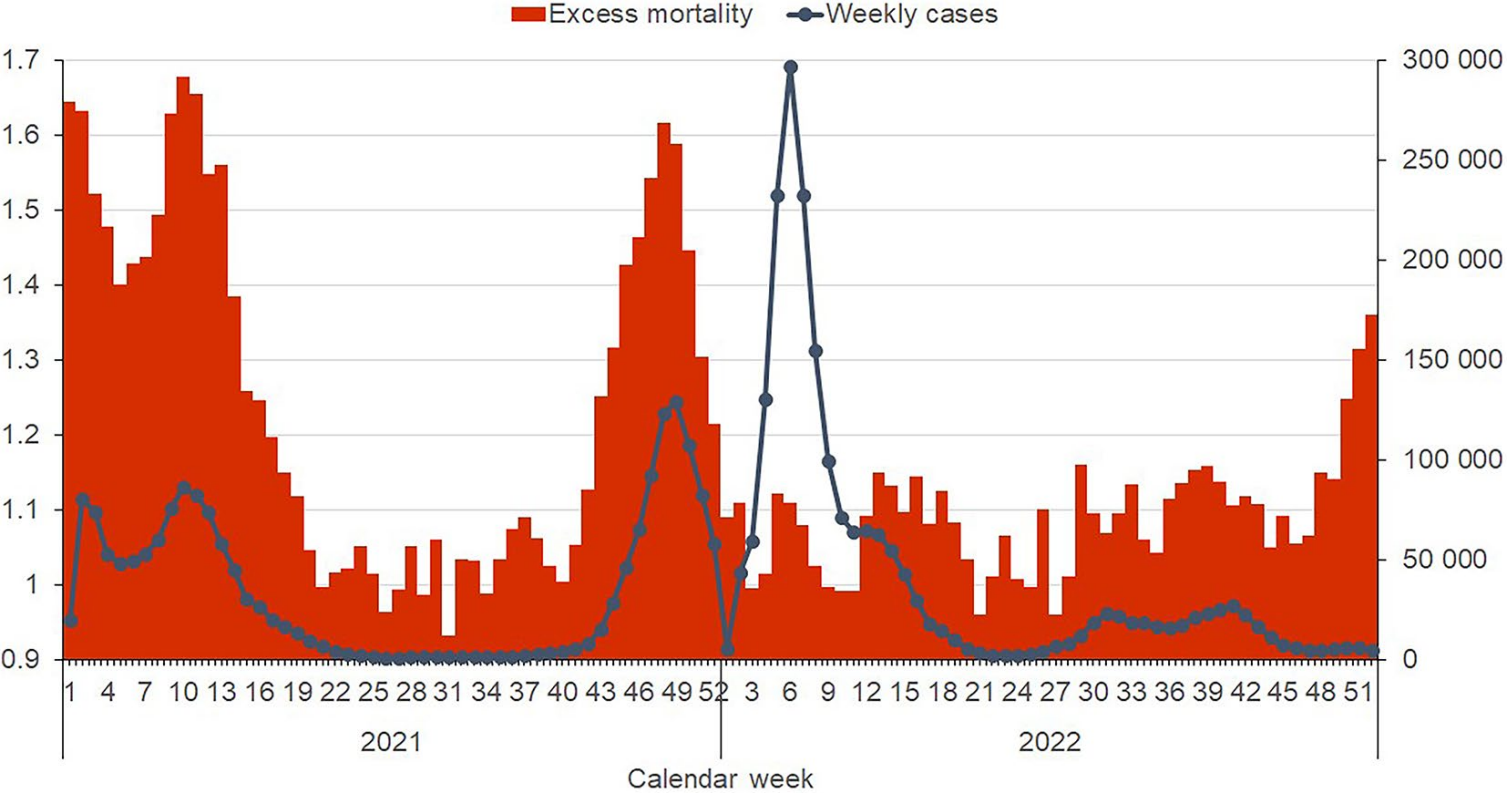
Excess mortality with weekly confirmed cases. Excess mortality is shown as a proportion of mortality during the reference period (2016–2019) along with smoothed weekly confirmed cases. Excess mortality mirrored case numbers in 2021, whereas the Omicron wave in early 2022 saw a dramatic increase in case numbers while excess mortality stayed near baseline levels.

Excess mortality was used as a comprehensive measure of the overall impact of the pandemic on mortality. In addition to confirmed deaths from COVID-19, it also includes deaths from other causes attributable to the pandemic situation including lack of access to healthcare or delayed preventive care. The Czech Republic experienced two peaks of excess mortality in 2021; between January to April and November to December (Fig. 5). In the first period, excess mortality peaked at 67.8% (22.9 vs. 38.4 deaths per 100,000) above baseline, corresponding to the rise in cases of the Alpha variant. The rise in excess mortality can be attributed to the virulence of the Alpha variant as well as low vaccine coverage in the Czech Republic. By the end of March 2021, only 14.5% of adults had received at least one dose of a vaccine.

Vaccinations were administered to at-risk adults from the 16th of January 2021 using a severity score to prioritise people. Elderly people aged 80+ as well as those aged 70–79 were vaccinated first, along with healthcare workers directly working with COVID-19 patients, as well as some social workers and elderly home carers. Employees enabling the operation of critical infrastructure such as emergency services, workers in the energy sector or crisis management teams were also initially prioritised. From the 19th of March people with chronic illnesses such as malignant disorders, diabetes, heart or lung disease, as well as some education workers had also begun receiving vaccinations. By the end of March, more than 514,000 people were vaccinated with two vaccine doses. Once sufficient quantities of vaccines had arrived in May and June, the registration for vaccination was opened to all adults.

The second peak in November 2021 saw excess mortality peak at 61.6% (32.3 vs. 20.0 deaths per 100,000). Since December 2021 the excess mortality rate rapidly declined due to the introduction and spread of the more infectious but less virulent Omicron variant, along with increasing primary course and booster vaccine uptake. By the end of the year 2021 up to 73.0% of adults had finished the primary vaccination course and 27.9% had received their first booster, with vaccine breakthrough infections or reinfections accounting for almost all new cases. As the daily case numbers increased past 40,000 in February 2022, excess mortality decreased to 6%, below the European average of 8%, as the dominance of the less virulent Omicron variant resulted in fewer deaths.

### Variant discrimination PCR

**Fig. 6:**
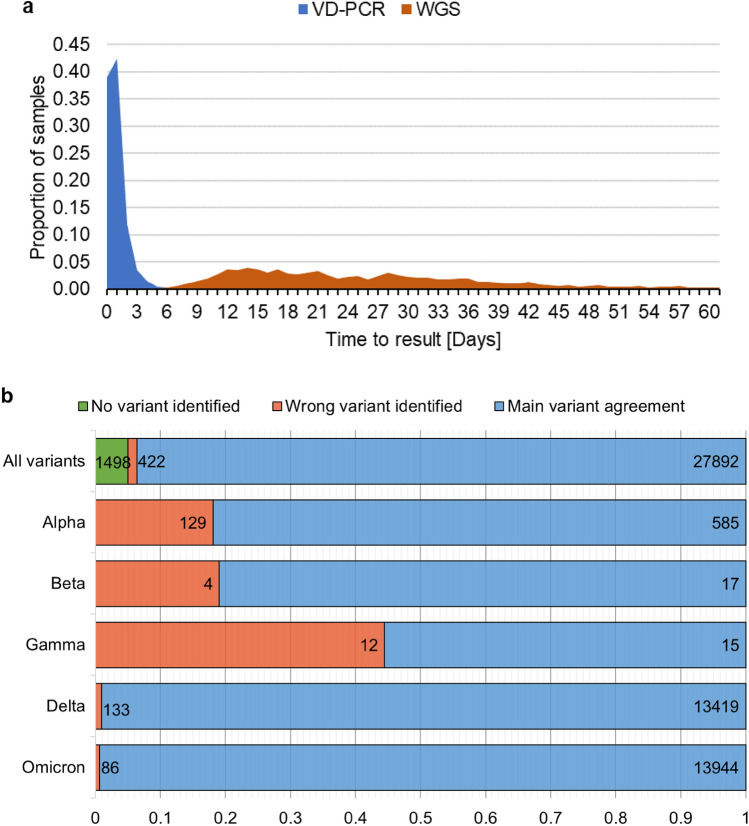

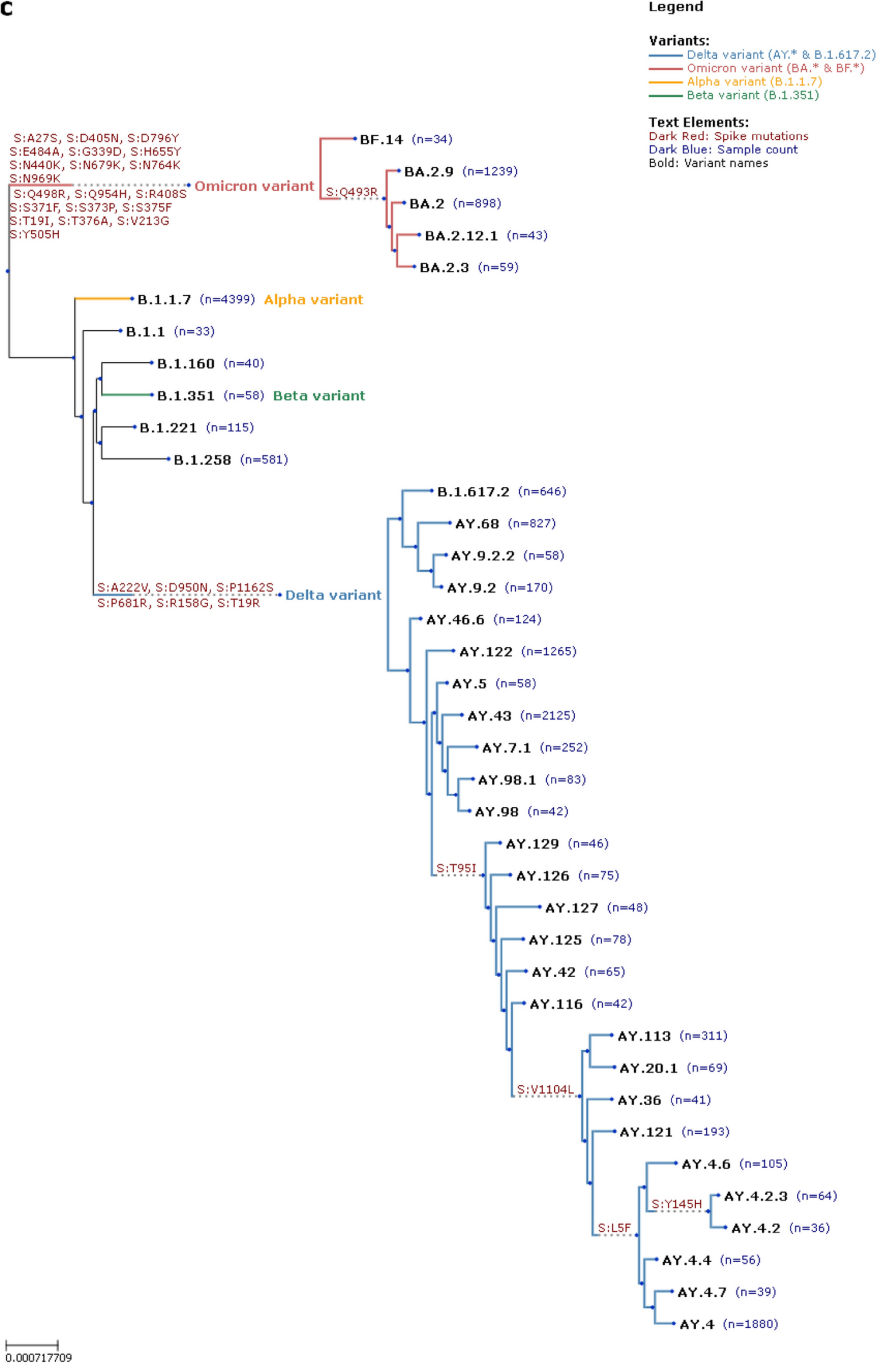
Comparison of VD-PCR and WGS surveillance. (**a**) VD-PCR results were available much sooner than WGS results, which had an inconsistent turnaround time. (**b**) The plot shows the number of samples analysed by VD-PCR assays stratified by the variant determined by WGS and the agreement of identified variants. (**c**) Emergence of novel variants in 2021 and 2022 saw dramatic increases in the number of novel mutations (show in red) as the Delta and Omicron variant emerged, resulting in increasing phylogenetic heterogeneity.

The genomic surveillance system initially scaled up slower than necessary during the Alpha wave, and VD-PCR was used to provide approximate variant identification (Fig. 6). Some testing facilities were also able to detect the Alpha variant by an N gene PCR cycle threshold (Ct) shift as they used PCR primers sensitive to N gene mutations. This was before kits enabling the targeted detection of mutations were widely available. The data from 1,159,145 VD-PCR assays were gathered, of which 29,812 samples also had whole genome sequences.

Mirroring the funding period, testing was greatly imbalanced temporally, with the vast majority (N = 1,068,792) of tests being performed during the transition from Delta to Omicron between October 2021 and March 2022 and only 90,353 tests performed in the remaining 18 months. In the vast majority of cases (93.6%) the results of the VD-PCR assay agreed with the main variant determined by WGS. Only in 422 (1.4%) cases did VD-PCR assays identify incorrect variants, with the remaining 1498 (5.0%) assays not providing enough data to determine any variant (Fig. 6b).

Variant identification was most accurate at discriminating Omicron and Delta variants with 99.4% and 99.0% of cases in agreement with WGS results, respectively. These variants pushed the overall average for VD-PCR accuracy higher, as Omicron and Delta samples accounted for 47.1% and 45.5% of the samples, respectively. The first half of 2021 saw the co-circulation of four variants of concern, necessitating their rapid identification in samples. Of the variants identified as Alpha by VD-PCR, 18.1% (N = 129/714) were false identifications. For Beta and Gamma variants this percentage was 19.0% (N = 4/21) and 55.5% (N = 15/27) respectively; however, the number of samples analysed by WGS was low. Similarly, pre-alpha variants were not identified due to the low usage of the VD-PCR assay at the time. Of all samples tested by VD-PCR, 39.1% had their results available on the same day as the collection date. This percentage increased to 81.5% within 48 h.

### Sequencing coverage

The minimal sequencing target set by the ECDC enables the detection of a variant circulating in 2.5% of cases, while the optimal recommended sequencing coverage enables the detection of a variant circulating in 1% of cases. The minimal ECDC sequencing target was met in 44 (41.5%) out of 106 weeks, whereas the optimal level was only achieved in a single week (week 2, 2022) (Fig. 1). The median time from the date of collection to the date of publication of the sequence in GISAID was 25 days, with 1.2 and 19.6% of samples sequenced within 7 and 14 days, respectively, over the two-year period.

Between January and March 2021, the number of cases per million people in the Czech Republic was 3.7 times higher than the European average (875 vs. 236), coinciding with the introduction of the more infectious Alpha variant^12^. Due to the gradual development of the sequencing network, the sequencing coverage during this period was below any ECDC recommended level. The highest intensity of sequencing was during the transition from Delta to Omicron variant dominance, from October 2021 to April 2022, with more than 3,000 samples sequenced monthly. The ECDC minimal sequencing coverage was consistently achieved between the 6th of September 2021 and the 15th of May 2022, a period of record case numbers in the Czech Republic. The sequencing centres struggled to scale their sequencing capacity further, with the 1,400 sample-per-week national target being reached once in the two-year period, while overall achieving the ECDC-recommended minimum level of surveillance in fewer than half of the studied months.

## Discussion

Some of the strictest public health measures of the pandemic were implemented between January and March 2021, when the Czech Republic detected more than 10,000 daily confirmed infections. This was caused by rapid spread of the more virulent Alpha variant. At a time when fewer than 15.0% of adults had received a single vaccine dose, many lives may have been saved by implementing pandemic precautions sooner, ensuring enforcement of restrictions and actively promoting the vaccination campaign. In this period of low vaccine availability, postponing the application of the second dose to 5 weeks after the first dose and prioritising vaccinating as many people as possible with a single dose may have decreased morbidity and mortality^14^.

When comparing pandemic restrictions in the Czech Republic with those in neighboring countries, Germany and Austria implemented stricter measures earlier, peaking in January and February 2021 as quantified by the stringency index^15^. In contrast, the Czech Republic, Poland, and Slovakia adopted less stringent and more gradual measures, which coincided with higher peaks in cases and excess mortality during the rapid spread of the Alpha variant^16–20^. These findings suggest that implementing stricter restrictions sooner to reduce peak case numbers could improve patient outcomes and reduce mortality. Advanced modelling in the Czech Republic has shown that delaying lockdowns even by several days may have dramatically increased the number of additional confirmed cases^20^. Participation in in-person voting during the October 2021 general election may have also accelerated the rate of confirmed infections and hospital admissions^21^. Genomic surveillance data provided critical insights that directly informed public health interventions. The detection of the B.1.1.7 (Alpha) variant led to the implementation of enhanced travel restrictions and targeted testing, which may have reduced transmission or mitigated excess mortality in early 2021. With the subsequent emergence of the Delta variant, which exhibited novel virulence and immune escape mutations as identified by molecular surveillance, public health efforts shifted toward accelerating vaccination uptake. Widespread vaccination not only reduced severe disease outcomes but was also integrated into reopening strategies, with access to large gatherings and public events increasingly contingent on vaccination status, reinforcing its role in pandemic management.

Region specific lineages such as AY.113 and AY.20.1 circulated from September 2021 during the delta wave and were eventually outcompeted by Omicron in January 2022. Their spread remained geographically localized within regions likely due to the combination of the implementation of restrictions from September 2021 and due to the high genomic variability of other circulating variants outcompeting them outside of the geographic niche. While there is evidence that these infections may have had a common origin, we do not have the epidemiological data to support this hypothesis. The majority of these cases occurred in Brno and the South Moravian Region which has a similar population density and vaccination rate to Prague, where these variants were very rarely detected. The observed dominance of the Delta variant in the November 2021 wave has been attributed to waning vaccine-induced immunity combined with the novel antigenic profile of the Delta variant, resulting in vaccine breakthrough infections^22,23^. Although delayed access to non-COVID-related healthcare and interruptions in screening programs undoubtedly affected the excess mortality rate, the Czech Republic’s rate was similar to that of neighboring Central European countries, where rates ranked among the highest in the world^24^.

While sample selection was handled by the ISIN algorithm in order to reduce selection bias, some regional differences were noted in WGS coverage, with low numbers of sequenced samples in the first half of 2021 in Karlovy Vary, Usti nad Labem, Liberec and Zlin regions resulting in extrapolated conclusions about variant distribution, likely due to reduced access to healthcare. The data showed that the ECDC-recommended minimal genomic surveillance was only reached in the Delta and Omicron waves once the sentinel surveillance system and sequencing network were fully established. Nonetheless, genome sequences were usually older than two weeks by the time they were published. The time required to determine the lineage by sequencing limited the ability to utilise sequence data to set epidemiological restrictions. Therefore VD-PCR assays were used to rapidly detect variants on a wide scale. The high rate of inaccurate variant prediction by VD-PCR for Alpha, Beta and Gamma variants stemmed from the use of testing kits with unsuitable mutation compositions compared to the recommendations made by the NRL. Mutation composition was not mandated and during 2021 when mutation testing kits were being rapidly developed, they were often subject to supply constraints. An incorrect variant prediction could be corrected in a minimum of 7 days by WGS, at which point any variant-specific epidemiological restrictions such as contact tracing and longer isolation would need to be enforced, subject to regional hygiene station capacity. WGS also facilitated the tracking of regional infection clusters for which targeted public health responses could be used. Moreover, the cost-effective amplicon sequencing protocols and rapid data sharing via platforms like GISAID were pivotal in adapting reagents to novel mutations and preventing regional blind spots in virus circulation, reinforcing the need for coordinated surveillance networks such as COG-CZ.

To enhance preparedness for future outbreaks, our main recommendation is to establish and support a national surveillance network (COG-CZ) that would coordinate molecular surveillance and collaborate with government institutions. This would involve continuous investment in advanced sequencing technologies and optimizing logistical networks between sentinel laboratories and sequencing centres to reduce turnaround times from sample collection to data reporting. The surveillance network should develop centralised automated sample selection, and provide rigorous training and best practices, to ensure rapid, consistent, and high-quality data publication. The surveillance network must be integrated into national pandemic preparedness plans and supported by sustained investments in surveillance capacity, including strengthening regional facilities, ongoing staff training and transparent financial planning, which is critical for maintaining readiness between outbreaks. Finally, integrating genomic data into public health decision-making, such as through real-time modeling and transparent communication with the public can guide targeted interventions and strengthen public trust in response efforts.

## Supplementary Information

Supplementary Material

## Data Availability

Confirmed infection data is publicly available online (https://onemocneni-aktualne.mzcr.cz/covid-19). Results of VD-PCR assays may be requested from Institute of Health Information and Statistics of the Czech Republic (IHIS/UZIS) (https://www.uzis.cz/index.php?pg=kontakt--zadosti-o-data-analyzy). GISAID metadata used is available for registered users using the sample set ID gisaid.org/EPI_SET_231101rf.

## References

[1] Ministry of Health of the Czech Republic [Internet]. [cited 2023 Nov 10]. Available from: https://onemocneni-aktualne.mzcr.cz/covid-19.

[2] Institute of Health Information and Statistics of the Czech Republic. Podání žádosti O export, Analýzu Dat Z NZIS [Internet]. [cited 2023 Nov 10]. Available from: https://www.uzis.cz/index.php?pg=kontakt--zadosti-o-data-analyzy.

[3] Shu, Y. & McCauley, J. GISAID: Global initiative on sharing all influenza data—from vision to reality. *Eurosurveillance* **22**(13), 30494. 10.2807/1560-7917.es.2017.22.13.30494 (2017). 28382917 10.2807/1560-7917.ES.2017.22.13.30494 PMC5388101

[4] Rambaut, A. et al. A dynamic nomenclature proposal for SARS-COV-2 lineages to assist genomic epidemiology. *Nat. Microbiol.* **5**(11), 1403–1407. 10.1038/s41564-020-0770-5 (2020). 32669681 10.1038/s41564-020-0770-5 PMC7610519

[5] Quick, J. NCoV-2019 Sequencing Protocol V3 (locost) [Internet]. protocols.io; 2020 [cited 2023 Nov 10]. Available from: https://www.protocols.io/view/ncov-2019-sequencing-protocol-v3-locost-bp2l6n26rgqe/v3.

[6] Mapleson, D., Drou, N. & Swarbreck, D. Rampart: A workflow management system for de novo genome assembly. *Bioinformatics* **31**(11), 1824–1826. 10.1093/bioinformatics/btv056 (2015). 25637556 10.1093/bioinformatics/btv056 PMC4443680

[7] Ewels, P. A. et al. The nf-core framework for community-curated bioinformatics pipelines. *Nat. Biotechnol.* **38**(3), 276–278 (2020). 32055031 10.1038/s41587-020-0439-x

[8] Li, H. & Durbin, R. Fast and accurate long-read alignment with Burrows-Wheeler transform. *Bioinformatics* **26**(5), 589–595. 10.1093/bioinformatics/btp698 (2010). 20080505 10.1093/bioinformatics/btp698 PMC2828108

[9] Garrison E, Marth G. Haplotype-based variant detection from short-read sequencing. arXiv preprint https://arxiv.org/abs/1207.3907 (2021).

[10] Guidance for representative and targeted genomic SARS-COV-2 monitoring [Internet]. 2021 [cited 2023 Nov 10]. Available from: https://www.ecdc.europa.eu/en/publications-data/guidance-representative-and-targeted-genomic-sars-cov-2-monitoring.

[11] COG-CZ. Reports [Internet]. [cited 2023 Nov 10]. Available from: https://virus.img.cas.cz/report.

[12] Mathieu, E., Ritchie, H., Rodés-Guirao, L., Appel, C., Giattino, C., Hasell, J. et al. Coronavirus pandemic (COVID-19) [Internet]. [cited 2023 Nov 10]. Available from: https://ourworldindata.org/coronavirus (2020).

[13] European Centre for Disease Prevention and Control. Covid-19 Vaccine Tracker: European Centre for Disease Prevention and Control [Internet]. [cited 2023 Nov 10]. Available from: https://vaccinetracker.ecdc.europa.eu/public/extensions/COVID-19/vaccine-tracker.html#uptake-tab.

[14] Berec, L. et al. Importance of vaccine action and availability and epidemic severity for delaying the second vaccine dose. *Sci. Rep.* **12**(1), 7638. 10.1038/s41598-022-11250-4 (2022). 35538118 10.1038/s41598-022-11250-4 PMC9086670

[15] Hale, T. et al. A global panel database of pandemic policies (Oxford Covid-19 Government response tracker). *Nat. Hum. Behav.* **5**(4), 529–538. 10.1038/s41562-021-01079-8 (2021). 33686204 10.1038/s41562-021-01079-8

[16] Karlinsky, A. & Kobak, D. The World Mortality Dataset: Tracking excess mortality across countries during the COVID-19 pandemic. *Elife* **10**, e69336. 10.1101/2021.01.27.21250604 (2021). 34190045 10.7554/eLife.69336 PMC8331176

[17] Liu, H. et al. The basis of a more contagious 501y.V1 variant of SARS-COV-2. *Cell Res.* **31**(6), 720–722. 10.1038/s41422-021-00496-8 (2021). 33893398 10.1038/s41422-021-00496-8 PMC8063779

[18] Klempt, P. et al. Distribution of SARS-COV-2 lineages in the Czech Republic, analysis of data from the first year of the pandemic. *Microorganisms* **9**(8), 1671. 10.3390/microorganisms9081671 (2021). 34442750 10.3390/microorganisms9081671 PMC8397935

[19] Brejová, B. et al. A sars-COV-2 mutant from B.1.258 lineage with ∆H69/∆V70 deletion in the spike protein circulating in Central Europe in the fall 2020. *Virus Genes* **57**(6), 556–560. 10.1007/s11262-021-01866-5 (2021). 34448987 10.1007/s11262-021-01866-5 PMC8390540

[20] Berec, L. et al. Delays, masks, the elderly, and schools: First covid-19 wave in the Czech Republic. *Bull. Math. Biol.* **84**(8), 75. 10.1007/s11538-022-01031-5 (2022). 35726074 10.1007/s11538-022-01031-5 PMC9208712

[21] Palguta, J., Levínský, R. & Škoda, S. Do elections accelerate the COVID-19 pandemic?. *J. Popul. Econ.* **35**(1), 197–240. 10.1007/s00148-021-00870-1 (2021). 34548754 10.1007/s00148-021-00870-1 PMC8446183

[22] Gangavarapu, K. et al. Outbreak.info genomic reports: Scalable and dynamic surveillance of SARS-COV-2 variants and mutations. *Nat. Methods* **20**(4), 512–522. 10.1038/s41592-023-01769-3 (2023). 36823332 10.1038/s41592-023-01769-3 PMC10399614

[23] Kliegr, T. et al. Can variants, reinfection, symptoms and test types affect COVID-19 diagnostic performance? A large-scale retrospective study of AG-RDTS during circulation of Delta and Omicron variants, Czechia, December 2021 to February 2022. *Eurosurveillance* **28**(38), 2200938. 10.2807/1560-7917.es.2023.28.38.2200938 (2023). 37733239 10.2807/1560-7917.ES.2023.28.38.2200938 PMC10515498

[24] Wang, H. et al. Estimating excess mortality due to the COVID-19 pandemic: A systematic analysis of COVID-19-related mortality, 2020–21. *Lancet* **399**(10334), 1513–1536 (2022). 35279232 10.1016/S0140-6736(21)02796-3 PMC8912932

