# Supplementary material for: Developing molecular surveillance of SARS-CoV-2 in the Czech Republic (2021–2022)
Source: Sci Rep. 2025 Jun 4;15:19690. doi: 10.1038/s41598-025-01074-3 (PMC12137580; doi:10.1038/s41598-025-01074-3)
Supplement: Supplementary file 1 — Supplementary Material [file 41598_2025_1074_MOESM1_ESM.pdf]

Title: Developing molecular surveillance of SARS-CoV-2 in the Czech Republic (2021–2022)

Authors: Timotej Šúri[1,2,3] (https://orcid.org/0009-0000-5402-8525), Lucie Pfeiferová[2,3] (https://orcid.org/0000-0003-1089-0329), Matěj Bezdíček[4] (https://orcid.org/0000-0002-5833-8325), Jan Svatoň[4,5](https://orcid.org/0000-0003-1817-305X), Vladimír Hampl[6] (https://orcid.org/0000-0002-5430-7564), Karel Berka[7] (https://orcid.org/0000-0001-9472-2589), Helena Jiřincová[1] (https://orcid.org/0000-0002-0566-2711), Martina Lengerová[4] (https://orcid.org/0000-0001-8739-9998), Martin Kolísko[8] (https://orcid.org/0000-0003-0600-1867), Alexander Nagy[1,9] (https://orcid.org/0000-0002-0512-8746), Ruth Tachezy[6] (https://orcid.org/0000-0001-7689-9727), on behalf of the Czech COVID-19 Genomics Consortium[†], Michal Kolář[2*] (https://orcid.org/0000-0002-4593-1525), Jan Pačes[2,3*] (https://orcid.org/0000-0003-3059-6127).

Affiliations

1. National Institute of Public Health, Prague, Czech Republic

2. Institute of Molecular Genetics of the Czech Academy of Sciences, Prague, Czech Republic

3. University of Chemistry and Technology, Prague, Czech Republic

4. Department of Internal Medicine – Hematology and Oncology, University Hospital Brno and Faculty of Medicine, Masaryk University, Brno, Czech Republic

5. CEITEC Masaryk University, Brno, Czech Republic

6. Faculty of Science, Charles University - BIOCEV, Prague, Czech Republic

7. Department of Physical Chemistry, Faculty of Science, Palacký University in Olomouc, Olomouc, Czech Republic

8. Biological Center of the Academy of Sciences of the Czech Republic, České Budějovice, Czech Republic

9. State Veterinary Institute, Prague, Czech Republic

† The members of the COG-CZ consortium are acknowledged at the end of the article

* These authors contributed equally to this work and share last authorship.

Supplementary information

Supplementary figure 1 *Geographic distribution of sequencing centres (Supplementary figure 1a) and a map of Czech regions (Supplementary figure 1b). Sequencing centres were concentrated in populous cities and were connected to a diffuse network or regional hospitals and healthcare centres. Each sequencing centre covered one or two regions, each covering approximately one million people. Figure was generated using the Google Maps Platform – Static Map (Version 11 Mar 2024 https://mapsplatform.google.com/).*

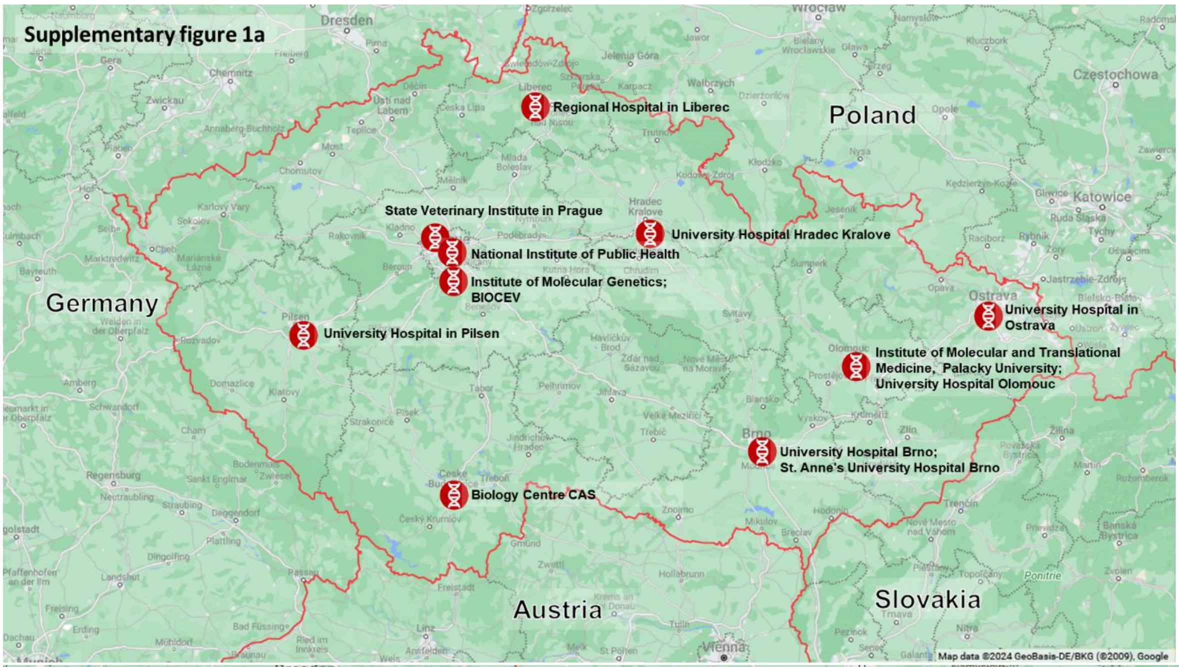

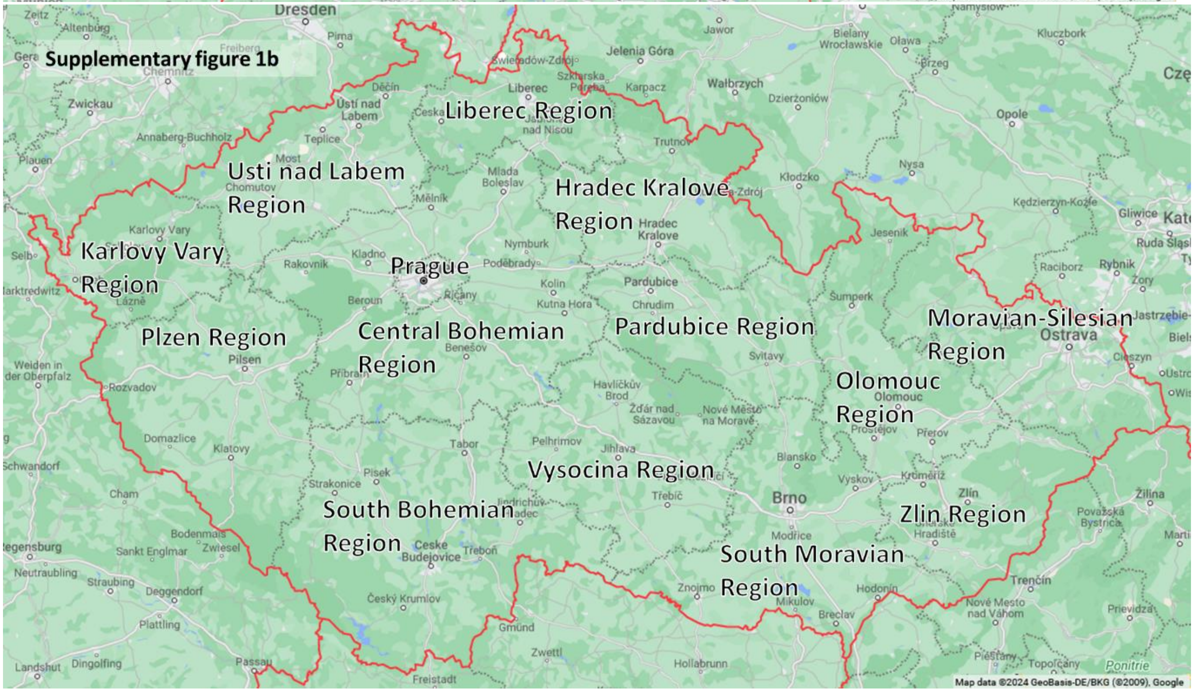

Supplement 2 *Cultural and epidemiological significance of some Czech regions. Border regions and major population centres were significant in the monitoring of import of new variants.*

Prague has the highest population density in the country and is a major cultural and economic hub that attracts international tourism. It also hosts the Czech Republic's main international airport, which handles around 10 million passengers annually. The Central Bohemian region surrounds Prague and has a mixture of rural and urban towns; it is the most populous region with over 1.4 million residents. The Moravian-Silesian region is also one of the most populated regions, with over 1.1 million residents and forms a key link in trade and transportation as it borders Poland. Brno is the regional capital of South Moravia, a major agricultural centre known for its wine production and its developed tech

sector. The Karlovy Vary region is famous for its spa towns and mineral springs and relies heavily on tourism.

Supplement 3 *Most commonly used kits for variant discrimination assays in the Czech Republic. Differences in kits and their varied usage affected the ability of some laboratories to identify particular variants.*

Many samples were tested using one of three mutation detection kits from different manufacturers, though kits by different manufacturers were available. Many laboratories used the combined diagnostic and variant discriminatory kit DBdirect™ RT-PCR (Diana Biotechnologies) testing for spike protein mutations E484K, L452R, and Y505H. This kit was coincidentally sensitive to N gene mutations, causing the Alpha-variant-specific N gene CT shift. The Allplex™ SARS-CoV-2 Variants Assay (Seegene) was also used, testing for E484K, Del69-70, N501Y, K417N/T, L452R, and W152C mutations. Additionally, the PowerChek™ SARS-CoV-2 S-gene Mutation Detection Kit (Kogene Biotech) was also recommended as it enabled the detection of A570D, E484K, N501Y, L452R, P681R, and E484Q mutations.

Supplement 4 *Quality criteria for WGS raw data and criteria for data publication. The parameters are based on the criteria by Robert Koch Institute and COVID-19 Genomics UK.*

1. Quality criteria for raw data

Supplementary table 1 lists quality criteria for raw data of the frequently used Illumina and Oxford Nanopore (ONT) technologies. When using alternative sequencing technologies (e.g., MGI, Ion Torrent, PacBio), use comparable criteria. In each sequencing experiment, at least one negative control sample must be present to detect possible contamination during processing.

Supplementary table 1. Quality criteria for raw data of Illumina and Oxford Nanopore (ONT) technologies.

Quality parameter	*Illumina*	*ONT*
Length of acceptable reads [1]	≥ 30 bp	≥ 200 bp
Average PHRED quality of acceptable reads	≥ 20	≥ 10

Note 1: When using amplicon-based protocols, adjust the length of acceptable reads with regard to the expected fragment lengths in order to exclude primer dimers.

2. Quality criteria for reconstructed genome sequences

Supplementary table 2 lists the quality criteria to be achieved for reconstructed SARS-CoV-2 genome sequences regardless of the sequencing technology used. The quality of the underlying raw data must be guaranteed (see section 1). Undesirable sequences or sequence regions such as contaminations (e.g., human sequences), sequencing adapters (even partial) and, in the case of amplicon-based protocols, primer and primer dimer sequences should be excluded prior to variant calling and data deposition. The reconstructed genome sequences must be reported in IUPAC nucleotide code (https://www.bioinformatics.org/sms/iupac.html).

Supplementary table 2. Quality criteria for reconstructed SARS-CoV-2 sequences

Quality parameter	*Threshold value / action*

Identity to NC_045512.2 [2]	≥ 90 %
Proportion of N's in the reconstructed genome	≤ 5 %
Minimum local sequencing depth without filtering of PCR and optical duplicates [3]	20 x
Minimum local sequencing depth after filtering of PCR and optical duplicates [3]	10 x
Informative allele frequency [4]	≥ 90 %
Frameshift mutations [5]	Manual inspection

Note 2: The percent identity refers to the total length of the reference sequence NC_045512.2 (https://www.ncbi.nlm.nih.gov/nuccore/1798174254). Only matching aligned informative bases (A, T, G, C) are considered identical.

Note 3: Positions in the reconstructed genome that are covered by fewer than 20 reads must be masked with N. If PCR and optical duplicates have been removed, the minimum local sequencing depth can be reduced to 10 reads.

Note 4: Informative positions (A, T, G, C) must be supported by at least 90 % of the aligned reads or, in the case of model-based basecalling (e.g., ONT), guaranteed by comparable means. Otherwise, less informative placeholders must be used according to the IUPAC nucleotide code (e.g., R, Y, N). Positions affected by strand bias should be reported as N.

Note 5: The validity of genomic variations that lead to frame shifts within a coding gene should be manually inspected by carefully examining the alignment of the affected positions, e.g., to avoid misreadings of homopolymeric genome regions.

Supplementary table 3 (to Figure 1) *Public health restrictions introduced throughout the pandemic. The introduction of new restrictions or their removal was in response to changing case numbers.*

In an effort to flatten pandemic waves, the Czech government mandated a variety of restrictions over the two-year period. Time points for newly implemented restrictions are in red, loosened restrictions are in green, and the progress of the vaccination campaign is in yellow.

**Date**	**Public health response**
Active restrictions prior to 1 January 2021	State of emergency declared, gatherings limited to 2 people, weddings and funerals to 15, and schools closed for those older than 2nd-year elementary school.<br>Bars, restaurants sports centres and cultural centres closed, ski slopes closed, night curfew after 9 pm, a negative antigen test or RT-PCR is required for travel from abroad, limits on entry of foreign nationals.<br>21-day isolation and household quarantine following confirmed infection.
01 January 2021	Restrictions on free movement, sales and provision of services, operations of public administration

16. January 2021	First vaccines available for high-risk adults (Comirnaty)
01 March 2021	Mandatory respirator wearing anywhere in public, ban on any non-essential travel or travel between administrative regions
03 March 2021	Mandatory workplace antigen testing every 7 days, except if <90 days post-infection or >14 days post full course vaccination. Isolation and household quarantine following confirmed infection reduced to 14 days.
08 March 2021	Work duty ordered for healthcare workers
12 April 2021	End of the state of emergency
24 April 2021	Mandatory once-weekly testing in higher education, exceptions for vaccinated or recovered students
10 May 2021	All retailers reopen, end to mandatory outdoor mask-wearing outside, all students return to school full-time
24 May 2021	Return of in-person teaching at universities and secondary schools, hotels open to guests
08 June 2021	End of mandatory mask-wearing, sports and cultural centres open to vaccinated visitors
21 June 2021	30% uptake of primary vaccination course in adults
01 July 2021	Increased capacity for sports and cultural events
09 July 2021	International travellers are required to present proof of vaccination or a negative PCR test
01 September 2021	School year starts for kids under 18
08 October 2021	In-person parliamentary election voting starts
22 November 2021	Restrictions on vaccination in sports centres and cultural events
23 November 2021	70% uptake of primary vaccination course in adults

26 November 2021	Christmas markets are prohibited, bars and clubs closed after midnight, and sports and cultural events limited in capacity
26 November 2021	New state of emergency declared; work duty ordered for healthcare workers
30 December 2021	Retail services, catered services or any social clubs are obligated to prevent the gathering of persons less than 1.5 metres from each other
03 January 2022	30% uptake of booster dose in adults
17 January 2022	Testing twice per week in all workplaces, limitations on vaccination exemption
10 February 2022	Restrictions on all businesses, sports and cultural events lifted, except for mass events with a maximum 1000 seated guests for cultural events and 100 people for other mass events
05 May 2022	Almost all remaining pandemic restrictions lifted
Active restrictions as of 31st December 2022	Mandatory mask-wearing in healthcare institutions, 7-day isolation in case of confirmed infection

Supplement 5 *GISAID Data Availability. The full dataset used is available to all registered users.*

GISAID Identifier:

EPI_SET ID: EPI_SET_231101rf

doi: 10.55876/gis8.231101rf

All genome sequences and associated metadata in this dataset are published in GISAID's EpiCoV database. To view the contributors of each individual sequence with details such as accession number, Virus name, Collection date, Originating Lab and Submitting Lab and the list of Authors, visit 10.55876/gis8.231020ez

Data Snapshot

EPI_SET_231101rf is composed of 56,033 individual genome sequences.

The collection dates range from 2021-01-02 to 2022-12-31;

Data were collected in 1 countries and territories;

All sequences in this dataset are compared relative to hCoV-19/Wuhan/WIV04/2019 (WIV04), the official reference sequence employed by GISAID (EPI_ISL_402124). Learn more at https://gisaid.org/WIV04.

Supplement 6 *The full list of public health measures adopted by the Czech government throughout the pandemic in 2021 and 2022. Available at (https://vlada.gov.cz/en/media-centrum/aktualne/measures-adopted-by-the-czech-government-against-coronavirus-180545/).*

2021

- **All emergency measures will be extended** until 22 January. It includes restriction of free movement, restriction of sales and provision of services, restriction of the operations of public administration and implementation of emergency measures ensuring childcare for employees of certain professions.
- As of 11 January it is also possible to arrange individual consultations even in the case of **university students**, hold university entrance exams in groups of no more than ten people and hold higher vocational school entrance exams also in groups of no more than ten people.
- Possibility for clients of health insurance providers to **be tested for coronavirus with an antigen POC test free of charge** been extended until further notice.
- There is also new **emergency measures regulating visits to healthcare facilities and social care facilities**. The changes apply to acute in-hospital care, where it will now be possible to visit a patient in a hospital provided that at least FFP2 or KN95 class respirators are used (this does not apply to children up to two years old; a simple face mask is sufficient for children up to 15 years old). This measure applies until 22 January.
- As of 12 January, there is a **change at the situation in funeral services**. It is prohibited to cremate the bodies of persons who have died outside of the Czech Republic that were neither citizens of the Czech Republic nor had permanent residence within the Czech Republic.
- **As of 19 January, there are minor adjustments to the currently valid rules.** The most important change is the opening of retail shops with stationery, children's clothing and children's footwear. Vocational training and the acquisition of specific professional qualifications will also be permitted for selected professions.
- **As of 19 January**, measure allowing an initial medical examination and proof of ownership of a health certificate when commencing employment to be replaced with a sworn statement is approved.
- The **state of emergency** in the Czech Republic will now apply **until 14 February.**
- **Valid crisis measures are also extended** until 14 February.
- **As of 23 January**, childcare facilities for children up to three may now run on a daily basis. This measure is also to be in place until 14 February.
- As of 30 January, there is a change concerning **rules on the free movement of persons**. people must limit contacts with non-household members to what is strictly necessary.

- As of 30 January, it would be possible to **provide accommodation** only to persons who can prove it with a document about the purpose of the business trip from the employer or the customer of the services.
- **Sales of goods in markets, marketplaces and mobile establishments** have been completely excluded from the exceptions still in force.
- **Only the operation of those mobile stores that sell food and cosmetics,** toiletries and household products in municipalities where it is not possible to purchase these goods in another establishment remains allowed.
- The rules for the operation of cableways and ski lifts are **also being tightened**. Newly, the operation of cableways will be permitted only for ensuring supply or operation of critical infrastructure or for the needs of IRS.
- As of 30 January, **visits in healthcare facilities** in acute care wards are **also banned** again. Exceptions are during childbirth, for patients with limited legal capacity and visits to patients in terminal stage of an incurable disease and hospice patients.
- Changes are also in the rules for **general antigen testing**. With immediate effect, the interval between tests fully covered by health insurance is reduced from five to three days.
- As of 15 February, a n**ew state of emergency is declared**. It will last for 14 days until 28 February.
- As of 15 February, **restrictions on the business hours of public authorities and administrative bodies are revoked.**
- As of 27 February, **new state of emergency applies in the Czech Republic**. It will last **until 28 March.**
- As of 27 February, **existing crisis measures has been re-confirmed** and extraordinary measures from Ministry of Health has been extended.
- As of 1 March, in the built-up area of a municipality, **it will be mandatory to wear everywhere outside** as a minimum a surgical mask, respirator or other similarly effective item of protective equipment meeting the relevant standard.
- As of 1 Match, in the most high-risk places, such as shops or public transport, it will **no longer be possible to replace the respirator with a double surgical mask.**
- As of 1 March, the **exception for employees is cancelled.** They will only be able to take off protective equipment if they are alone in the workplace.
- As of 1 March, **employers must equip those employees who come into contact with other people with a sufficient number of items of protective equipment for each shift.**
- As of 1 March, it will be **forbidden to leave the district, or the City of Prague**, in which the person in question has permanent residence or domicile, without good reason, such as travel to work or to a doctor.
- As of 1 March, if **people own a holiday home, they may go to it** until the entry into force of the regulation, but it then will be considered as their place of residence for the duration of

the measure. In the event of an inspection, everyone must be able to justify travel outside their own district either by affidavit or by an appropriate confirmation.

- As of 1 March, **only necessary trips**, such as shopping, trips to the countryside and sports activities, will be allowed, but only under the condition of the presence together of just the members of a single household, in the municipality in which those persons have permanent residence or domicile.
- As of 1 March, the **number of exemptions from the ban on the presence of the public in work premises is reduced**. Exceptions no longer include, for example, laundries and dry cleaners, car repair shops and spare parts shops, textile material and textile haberdashery shops, locksmiths, stationery shops and shops with weapons and ammunition.
- As of 1 March, **day care for children under the age of three will also have to close down**, with the exception of a children's group at a medical or social services facility.
- As of 1 March, **full-time school attendance** will be suspended for pupils in the 1st and 2nd years of primary schools, including preparatory levels, special schools and one- and two-year practical schools. Kindergartens are also closed.
- As of 1 March, **primary schools, kindergartens and children´s groups at medical facilities or at institutions for institutional or protective education remain open**. Regulation requiring regional governor and mayor of Prague to provide childcare for employees of critical infrastructure and integrated rescue system is still in force.
- As of 2 March, **driving school exams will be banned**, and employees will be banned from eating directly at a personnel catering establishment.
- As of 3 March, **employers are required to ensure antigen test for their employees** that will be performed either by healthcare professionals or directly by the person being tested.
- As of 3 March, **employees will once again be able to use company canteens for direct consumption of food**. However, only one diner may be seated at one table, and if it is a long table, there must be at least a two-metre gap between the diners or a mechanical obstacle preventing the spread of droplets.
- As of 3 March, there is an adjustment to the **length of the ordered quarantine** for persons living in the same household with a person infected with SARS CoV-2 coronavirus where conditions for the isolation of the infected person from other household members cannot be met. In this case, the length of the quarantine is reduced from 21 days to 14 days.
- As of 4 March, **attendance of meetings** is added to the daytime curfew, and it is specified that persons attending such meetings must wear a respirator or a surgical face mask or a similarly effective certified respiratory protective device.
- As of 5 March, **employers must invite their employees to undergo the test**, and from 12 March 2021, they must not allow employees who have not been tested in the past seven days access to the workplace. In the case of smaller companies, i.e. from 50 to 249 persons, the deadlines are postponed to March 5, 8 and 15, respectively.
- As of 20 March, people who can prove that either they have undergone the Covid-19 disease in the last 90 days or have been completely vaccinated for more than 14 days will be exempt

from the obligation to submit a valid negative test for Covid-19 **when visiting patients in health-care facilities**.

- As of 20 March, the same rule will also apply for the **exception from the mandatory quarantine for clients of social service facilities** who have gone on leave outside of the complex or are accommodated in them.
- As of 22 March, people **can now go on trips to nature or to exercise on the territory of the entire district**. Now an exception to the ban on leaving the district will also apply to parents for contact with their minor children or visits of close persons for children in foster families or institutional or protective care.
- Measure **ordering the wearing of protective respiratory coverings** makes a change for officials and employees of the basic units of the integrated rescue system, which will also be able to use a mask or half-mask fulfilling all the technical conditions and requirements.
- As of 30 March, there is also **an expansion of mandatory testing of employees**. The ban on the presence of untested employees will also apply to workplaces of public employers who employ less than 50 people. Testing of employees has to start no later than 23 March.
- **As of 21April,** the activity of children's groups **will be permitted in the Karlovy Vary and the Hradec Králové Regions**. In the other regions, it will be permitted only for children who reached five years of age by 31 August 2020, or for providing childcare services to parents of selected professions and employers.
- As of 21 April, the **measure ordering the wearing of breathing protection** will be specified so that participants in entrance examinations for secondary schools will be able to use a medical mask or a certified protective equipment instead of a respirator.
- As of 24 April, **across-the-board testing will be launched at higher education institutions**, under conditions similar to those in the other segments with generally ordered testing. Exemptions apply to completely vaccinated persons and persons who recovered from COVID-19 in the last 90 days.
- As of 26 April, **secondary school pupils will be able to attend practical training**. Students of the last grades of higher education institutions will be allowed to start practical and clinical instruction. From the same date, further children will return to nursery schools in the Karlovy Vary and the Hradec Králové Regions.
- As of 26 April, **kindergartens and children's groups will open in the Plzeň region**.
- As of 3 May, **Body care services and animal care services** will be permitted under the condition that each staff member cares for one client at a time, clients keep at least two-meter distance from each other and every client shows a valid negative COVID-19 test result, or a proof of completed vaccination or of recovery from COVID-19 within the last 90 days.
- As of 3 May, in the Hradec Králové, the Plzeň, the Karlovy Vary, the Pardubice, the Liberec, the Central Bohemian Regions and Prague, **individual tours will be permitted in museums**, galleries, castles, chateaux and other historic or cultural structures. Their visitors will be obligated to use respirators.
- As of 3 May, pupils in the Hradec Králové, the Plzeň, the Karlovy Vary, the Pardubice, the Liberec, the Central Bohemian Regions and Prague will be **able to attend in-person instruction**

in the lower level of six-year and eight-year grammar schools, in the first four years of the eight-year conservatory education programme and in the upper primary schools, under the condition of rotating presence of classes.

- **As of 3 May, rules for testing school and school facilities staff will be relaxed so that preventive testing will be carried out only once a week. Also, those students who already go to school regularly will have to be tested only once a week. Newly returning school years will remain in the regime of two tests per week.**
- **As of 4 May,** children from the Karlovy Vary, Hradec Králové, Liberec, Pardubice, Plzeň and Central Bohemia Regions and the capital city of Prague **will be able to attend group consultations** or classes indoors or outdoors at basic art schools and leisure time centres.
- **As of 10 May, all retailers will be able to open**, the reduction of a range of products and markets will also end, the range of services will be significantly expanded, and cable cars will be able to start too.
- As of 10 May, **other groups of pupils and students** will return to schools for full-time classes.
- As of 10 May, it will **no longer be mandatory to wear outdoor protection** where it will be possible to keep the maximum number of two people on less than two meters.
- **As of 17 May, restaurants are allowed to open their outdoor seating**, provided the set rules are observed. The seating capacity at tables is restricted and all customers must demonstrate a COVID-19 negative test, vaccination or recovery within the last 90 days.
- As of 17 May, **meals and drinks may be consumed in marketplaces**.
- As of 17 May, **first cultural events are launched, outdoor ones for up to 700 seated spectators, at maximum filling 50 percent of the auditorium capacity,** and even those have to demonstrate a COVID-19 negative test, vaccination or recovery, and have to wear a breathing protection during the performance.
- As of 17 May, **zoos or botanical gardens can raise their capacity to a half of the maximum number of visitors**.
- As of 17 May, **restrictions on club, sports, dance and other organised events** has been partially lifted, enabling them to be attended by up to fifty persons outdoors or ten indoors, under the usual anti-epidemic conditions.
- As of 17 May, the **ban on visits in acute health care facilities is cancelled**. The visitors are subject to the same rules that apply to visitors in long-term care facilities or in hospices.
- As of 17 May, the **weekly rotating in-person attendance ended for the lower primary schools**. The rotating attendance is cancelled for upper primary schools and lower levels of multi-year grammar schools and conservatories in the Karlovy Vary, Hradec Králové, Liberec, Pardubice, Plzeň and Central Bohemian Regions and the City of Prague.
- **As of 18 May,** the **use of breathing protection is modified**. The exceptions will newly include sportspeople and exercising persons during their training, exercise, match, competition etc. including running and cycling, as well as customers of services providing care on the head and neck where the protective mask would prevent the use of that service.

- **As of 24 May, in-person learning will be reopened for higher education students in theoretical subjects and for all remaining secondary school students**. The weekly rotation of classes will end where it has been ordered up to now.
- As of 24 May, the **limits for the number of persons will increase in leisure learning centres**, hobby education facilities for children, at cultural, sports and club events indoors and outdoors, at congresses or on sports grounds.
- As of 24 May, the **limits will be cancelled for entry in indoor pavilions of zoos or botanical gardens** for individual tours, there will only be the limitation of letting in only 50 percent of the visitor capacity, 15 m^2 of indoor area per person and two-metre distancing.
- As of 24 May, the **restriction on accommodating guests in hotels and guest houses will be lifted**.
- As of 31 May, it will be possible to **renew countryside learning stays of schools**. the school has to notify the public health authority of the countryside stay, including the address and contact details of the responsible person on the site, and all participants must produce a valid COVID-19 negative test, certification of recovery from the disease or certificate of complete vaccination.
- As of 24 May, the **protection period of persons recovered from COVID-19 is extended from 90 to 180 days** and may be used in situations where it is required as one of the alternatives enabling an entry or provision of service. Also the first dose of vaccine received at least 22 days prior will be accepted.
- **As of 8 June, the <u>conditions for entry into establishments providing services and into sports or cultural institutions will be levelled out</u>.** Visitors will be allowed to produce all possible forms of proof of being COVID-19 negative, including a self-test carried out directly on the spot. Maximum 1,000 people inside or 2,000 outside are allowed.
- As of 8 June, **<u>pupils, students and teachers no longer have to use protective devices</u>** while seated during lessons or lectures. The same exception applies to employees at their workplace, if they are exclusively in the presence of their co-worker. The rules for wearing breathing protection are eased in all regions except for the South Bohemian, Liberec and Zlín Regions.
- As of 8 June, the **<u>conditions are modified for visits to health facilities and social care facilities</u>**: the validity of PCR tests is extended to seven days, and that of POC antigen tests to 72 hours, and tests performed by employers or schools will be recognised too.
- **As of 15 June, <u>stricter rules for wearing respiratory protection</u>** in schools and workplaces in the South Bohemia, Liberec and Zlín regions are abolished.
- As of 1 July, **maximum number of customers per establishment, or in zoos, museums and castles will increase.** Limit of the permitted capacity of swimming pools, spas and saunas will be increased to 75 per cent off the capacity.
- As of 1 July, the **obligation for hairdressers and pedicurists to serve in a one-on-one mode** and keep a register of customers will end.

- As of 1 July, the maximum number of persons allowed at leisure events for children and young people and for the organisation of social, dance, cultural or sporting events **will be increased to 500 indoors and 1,000 outdoors.**
- As of 1 July, the number of spectators allowed at concerts and other cultural, sporting and educational events will also be **increased to 5,000 outside and 2,000 inside**, and standing spectators will be allowed on the condition of one spectator per four metres of standing area.
- As of 9 July, the **recognition of infection-free status after the first dose of a two-dose vaccine will no longer apply in the Czech Republic**. Person will be considered infection-free only 14 days after their completed vaccination.
- As of 9 July, the **rules for entering the Czech Republic will be tightened.** Travellers low-risk countries will now have to fill in an arrival form and, if they are not 14 days after their completed vaccination, they will also have to prove themselves with a negative RT-PCR test. For countries with a low and medium risk of infection, it will be possible to replace the pre-entry test with a test taken within five days of their return.
- As of 9 July, **unvaccinated persons returning from abroad will now be barred from entering their workplace without proving a negative test** to their employer. It will be possible to go to work after returning from low- and medium-risk countries until a negative test is proven, but these employees will be required to wear at least FFP2 respirators at work.
- As of 9 July, in countries with a low and medium risk of infection, it will be possible to replace the test before entering the Czech Republic with a test taken within five days of return.
- At the same time, for unvaccinated persons, when returning from abroad, it will now be the case that employers will not be able to be admitted to the workplace without proving a negative test. In the case of return from low- and medium-risk countries, it will be possible to attend employment until proven by a negative test, but these employees will be required to wear at least FFP2 respirators in the workplace.
- As of 15 July, the **interval between the two doses of Pfizer/BioNTech's Comirnaty vaccine will be reduced** from the current 38–42 days to 21–23 days after the first dose.
- Up to 22 July, **organisers of mass events** with an expected attendance of more than 1,000 people have to notify the relevant regional hygiene station of the event without delay.
- As of 23 July, organisers of mass events have to notify the relevant regional hygiene station **no later than 5 days before the start of the event**.
- As of 15 November, the rules for visits to health and social care facilities will be tightened by reducing the validity periods to the mandatory 72 hours for PCR and 24 hours for RAT tests, while the option to self-test on entry will remain.
- As of 22 November, the emergency measures restricting retail and service operations will also change. The measure adds rules for ski resorts and ski lifts, where operators will have to check the validity of ONT (vaccination – past disease – test) when selling tickets and ski passes and then at least randomly at the lifts and ski lifts.
- As of 22 November, the conditions for organising mass events will also change. A major change is that in cultural, artistic and sporting events with an attendance of over 1,000 spectators the

antigen tests will no longer be recognised, and that a uniform limit of 1,000 people for indoor and outdoor events is set for social and similar public events.

- **A 30-day state of emergency was declared as of 26 November.**
- As of 26 November, catering establishments, bars, discos, casinos, etc., which will have to be newly closed between 10:00 pm and 04:59 am.
- As of 26 November, the operation of Christmas markets will be prohibited, with the exception of the sale of Christmas trees and carp and in shopping centres it will not be possible to eat directly in food courts.
- As of 26 November, with the exception of funerals, the maximum number of people allowed at leisure, cultural, sports, educational and other similar events will also be limited.
- For leisure activities for children and adults, such as various club, sports, cultural, dance, traditional and similar events and celebrations, the maximum number of participants is reduced from one thousand to 100 people.
- For visits to cultural performances, sports matches, etc., it will be possible to let a maximum of 1,000 spectators enter the auditorium, exclusively for sitting.
- As of 26 November, there is **ordered the work duty of doctors and healthcare professionals** who practice a non-medical healthcare profession as outpatient specialists, day care or inpatient care physicians in facilities that do not care for patients with covid-19.
- As of 29 November, the **rules for visits to reception and accommodation centers** are the same as for prisons or detention facilities. The visit will therefore have to be demonstrated by meeting the O-T-N conditions, while a valid RT-PCR test will be recognized. The same exceptions to this obligation apply, ie for children under 12, lawyers or officials.
- The current government regulation, which allows the help of Czech Army soldiers in managing the covid-19 epidemic in medical facilities, social care facilities and vaccination centres have been extended until 30 June 2022.
- As of 14 December, the rule on the use of respiratory protection when using cable cars is clarified so that the obligation to use a respirator will only apply to enclosed cabins.
- As of 14 December, the rules for PCR testing of pupils and staff in schools will be specified.
- As of 20 December, the rules of further procedure will be changed in the case of a positive case in the area testing of employees and self-employed persons.
- As of 30 December, in retail and services, the rule on compulsory establishment and customer separation and the rule that it is not possible to let more people into the establishment than there are seats inside the seat are abolished for music and dance clubs and discos.
- As of 30 December, catering services, music, dance, gaming and similar social clubs and discos, casinos and casinos are also abolished by the operator's obligation to actively prevent the gathering of persons at a distance of less than 1.5 meters from each other on the outside and inside of the establishment.

2022

- As of 3 January 2022, this measure will be amended so that the abolition of the obligation to actively prevent the gathering of people will also affect other establishments and services where it is still required, such as markets, swimming pools or cable cars.
- As of 3 January, wherever customers and clients are required to provide proof of vaccination or illness, it will introduce an obligation for operators to check the validity of certificates through the mobile application of the Ministry of Health čTečka.
- As of 3 January, the rules of how schools, their employees or pupils should proceed <u>in the event of a positive test case are changed</u>.
- As of 3 January, testing in schools will take place on Mondays and Thursdays.
- As of 4 January, all persons over the age of 18 who have had at least 5 months of completed vaccination will be able to register for the third dose.
- As of 11 January, the ordered isolation and quarantine will be reduced to at least five calendar days. To terminate the quarantine or isolation, the RT-PCR test will not be necessary, but persons with a positive antigen test result will be able to end their isolation immediately after receiving a negative result of a subsequent RT-PCR test.
- As of 11 January, people with COVID-19 symptoms will have to extend their five-day isolation and wait at least two days after the symptoms fade out. After a risk contact, quarantine will be ordered also to COVD-19 vaccinated and recovered persons.
- As of 17 January, <u>testing will take place again only once a week</u>, the first day of attendance. Testing will now also cover school staff and the exemption for vaccinated or covid-19 people will no longer apply.
- As of 17 January, all employees in both the public and the private sectors, as well as self-employed persons and agents of legal entities, if they come into contact with people outside their households, will have to **<u>get tested at their workplace twice a week</u>.**
- As of 31 January**,** the **rules for testing employees, the self-employed and members of corporate bodies will change** so that everyone who has had a positive RT-PCR test in the last 30 days and has ended up in isolation will have an exemption from testing.
- As of 31 January, the <u>students of higher vocational schools</u> will also be involved in the testing.
- From 27 January to 13 February, **visits to prison, pre-trial detention facilities and detention facilities will be banned** with precisely specified exceptions.
- As of 10 February, **restrictions and conditions on trade, services and leisure activities will be lifted**. Only the number of people at mass events will continue to be regulated.
- Until 19 February, the limits will remain at the current levels, i.e. a maximum of 1,000 seated spectators or guests at cultural performances, sporting events or congresses, and a maximum of 100 people at other mass events and leisure activities for children and young people.
- As of 15 February, the **<u>validity of certificates of completed vaccination</u> for persons over 18 years of age to a maximum of 270 days will apply** in the Czech Republic.

- As of 19 February, a limit of 500 spectators will apply where there is no seating, and auditoriums with a capacity of over 1,000 seats will be able to fill up to 50 per cent of the seating capacity above the 1,000-seat limit.
- As of 19 February, the **conditions for testing employees in health and social services** are also modified by narrowing the scope of the emergency measure from all providers of health and social services to providers of overnight and inpatient care and providers of social services in facilities of homes for persons with disabilities, homes for the elderly or homes with special regimes, providers of social services providing respite services in residential form and providers of social services in weekly residential or sheltered housing facilities, and the possibility of using so-called work quarantine is abolished.

Supplement 7 *Czech COVID-19 Genomics Consortium members that did not directly contribute to the paper.*

Jan Bartoš[10], Petr Brož[11], Vojtěch Bystrý[5], Martin Čech[12], Monika Čechová[13], Jiří Černý[14], Kateřina Chudějová[15], Iva Dolinová[16], Pavel Dřevínek[17], Edvard Ehler[2], Alena Fialová[1], Filip Franko[6], Viktor Furman[18], Zoltán Füssy[19], Markéta Gančarčíková[20], Alejandro Jiménez González[6], Marián Hajdúch[21], Blanka Hamplová[6], Václav Hejret[5], Petr Holub[22], Patrik Horna[3], Miluše Hradilová[2], Štěpánka Hrdá[6], Magdalena Jančářová[6], Michaela Jaroměřská[7], Eduard Ježo[23], Daniel Klimeš[24], Šárka Kocourková[2], Jana Kozáková[1], Martin Kracík[16], Jan Kubovčiak[2], Jana Fialová Kučerová[25], Jan Kynčl[1], Matej Lexa[13], Monika Liptáková[1], Jan Macháň[21], Barbora Macková[1], Marek Malý[1], Kateřina Matějová[26], Luděk Matyska[22], Hana Medová[2], Ondřej Moravčík[2], Jakub Mrázek[23], Serafim Nenarokov[8], Eva Niklova[1], Marian Novotný[6], Jaroslav Nunvář[6], Adam Obr[27], Hana Orlíková[1], Josef Pasulka[2], Helena Paszeková[18], Ingrid Poláková[6], Martin Pospíšek[6], Miroslav Ruda[22], Jana Šáchová[2], Eric Salomaki[8], Jeffrey Silberman[8], Radek Šíma[8], Branislav Šiška[21], Rastislav Slavkovský[21], Klára Sobotíková[6], Kateřina Štillerová[16], Viktor Stránecký[6], Alexander Tice[8], Boris Tichý[5], Markéta Tomková[28], Karolína Trachtová[5], Sebastian Cristian Treitli[6], Jaromíra Večeřová[1], David Vostřák[1], Jan Vrbský[29], Monika Wisniewska[8], Artsemi Yushkevich[7], Tomáš Zajíc[16], Martin Zmuda[30].

Affiliations

1. National Institute of Public Health, Prague, Czech Republic

2. Institute of Molecular Genetics of the Czech Academy of Sciences, Prague, Czech Republic

3. University of Chemistry and Technology, Prague, Czech Republic

4. Department of Internal Medicine – Hematology and Oncology, University Hospital Brno and Faculty of Medicine, Masaryk University, Brno, Czech Republic

5. CEITEC Masaryk University, Brno, Czech Republic

6. Faculty of Science, Charles University - BIOCEV, Prague, Czech Republic

7. Department of Physical Chemistry, Faculty of Science, Palacký University in Olomouc, Olomouc, Czech Republic

8. Biological Center of the Academy of Sciences of the Czech Republic, České Budějovice, Czech Republic

9. State Veterinary Institute, Prague, Czech Republic

10. Institute of Experimental Botany of the AS CR, Olomouc, Czech Republic

11. Bioxsys s.r.o., Prague, Czech Republic

12. Institute of Organic Chemistry and Biochemistry of the Czech Academy of Sciences, Prague, Czech Republic

13. Faculty of Informatics Masaryk University, Brno, Czech Republic

14. Czech University of Life Sciences, Prague, Czech Republic

15. Faculty of Medicine in Pilsen, Charles University, Plzeň, Czech Republic

16. Regional Hospital Liberec, a. s. , Liberec, Czech Republic

17. Second Faculty of Medicine – Charles University, Prague, Czech Republic

18. GHC Genetics, s.r.o., Prague, Czech Republic

19. University of South Bohemia in České Budějovice, České Budějovice, Czech Republic

20. University Hospital Hradec Králové, Hradec Králové, Czech Republic

21. Institute of Molecular and Translational Medicine, Olomouc, Czech Republic

22. Institute of Computer Science Masaryk University, Brno, Czech Republic

23. Public Health Institute Ostrava, Ostrava, Czech Republic

24. Institute of Health Information and Statistics of the Czech Republic, Prague, Czech Republic

25. Faculty of Medicine Masaryk University, Brno, Czech Republic

26. University Hospital Ostrava, Ostrava, Czech Republic

27. Institute of Hematology and Blood Transfusion, Prague, Czech Republic

28. Ludwig Cancer Research Oxford, University of Oxford, Oxford, United Kingdom

29. St. Anne’s University Hospital Brno, Brno, Czech Republic

30. Institute of Microbiology of the Czech Academy of Sciences, Prague, Czech Republic
